# Enhancement of Lodging Resistance and Lignin Content by Application of Organic Carbon and Silicon Fertilization in *Brassica napus* L.

**DOI:** 10.3389/fpls.2022.807048

**Published:** 2022-02-17

**Authors:** Yue Hu, Hafiz Hassan Javed, Muhammad Ahsan Asghar, Xiao Peng, Marian Brestic, Milan Skalický, Abu Zar Ghafoor, Hafsa Nazir Cheema, Fang-Fang Zhang, Yong-Cheng Wu

**Affiliations:** ^1^College of Agronomy, Sichuan Agricultural University, Chengdu, China; ^2^Key Laboratory of Crop Ecophysiology and Farming System in Southwest China, Chengdu, China; ^3^Department of Biological Resources, Agricultural Institute, Centre for Agricultural Research, ELKH, Martonvásár, Hungary; ^4^Department of Plant Physiology, Slovak University of Agriculture, Nitra, Slovakia; ^5^Department of Botany and Plant Physiology, Czech University of Life Sciences, Prague, Czechia

**Keywords:** lodging, lignin, organic carbon, silicon, rapeseed (*B. napus* L.)

## Abstract

This study was aimed to investigate the effects of organic carbon and silicon fertilizers on the lodging resistance, yield, and economic performance of rapeseed. Two cultivars, namely Jayou (lodging-resistant) and Chuannongyou (lodging-susceptible), were selected to evaluate the effects of various fertilizer treatments on rapeseed culm morphology, lignin accumulation, and their relationships with their lodging resistance indices. The results showed that both organic carbon and silicon fertilizer applications increased the plant height, basal stem diameter, internode plumpness, and bending strength of rapeseed in both the studied years. The bending strength was significantly and positively correlated with the lodging resistance index and lignin content. It was found that both organic carbon and silicon fertilizers had improved the activities of lignin biosynthesis enzymes (phenylalanine ammonia-lyase, 4-coumarate:CoA ligase, cinnamyl alcohol dehydrogenase, and peroxiredoxins) and their related genes to increase lignin accumulation in the culm, which ultimately improved the lodging resistance. At the same time, the thickness of the stem cortex, vascular bundle area, and xylem area was increased, and the stem strength was improved. The effect of silicon fertilizer was better than that of organic carbon fertilizer, but there was no significant difference with the mixed application of silicon fertilizer and organic carbon fertilizer. Similarly, silicon fertilizer increased the number of pods, significantly increased the yield, and improved the economic benefit, while organic carbon fertilizer had no significant effect on the yield. Therefore, we believe that organic carbon and silicon fertilizer can improve the lodging resistance of rape stems by improving the lignin accumulation and the mechanical tissue structure. Still, the effect of silicon fertilizer is the best. Considering the economic benefits, adding silicon fertilizer can obtain more net income than the mixed application of silicon fertilizer and organic carbon fertilizer.

## Introduction

Rapeseed is the main oil crop in China and one of the four largest oil crops in the world (Li et al., 2016). With the increasing demand of the edible oil market, higher requirements for rapeseed yield are put forward. Increasing planting density is an effective measure to increase yield (Zhang et al., 2019), especially in the southwestern mountainous regions of China, where arable land is limited. Still, excessive planting density often leads to weak stems that increase the risk of lodging (Ling et al., 2010). Therefore, we focused on expanding the lodging resistance of oilseed rape by different agronomic measures under certain conditions of high stand density, which could also adapt to mechanical harvesting while increasing yield (Kaack and Schwarz, 2001).

Lodging is a common problem faced in the production of different crops. Generally, it refers to the phenomenon of unrecoverable displacement of culms, which reduces yield and quality (Berry et al., 2004). The occurrence of lodging is affected by various factors, including high planting density and high nitrogen fertilization. Wang et al. (2015b), Zhang et al. (2017), and others such as natural disasters: heavy rains, strong wind, and pests and diseases also contributing to crop lodging occurrence (Cleugh et al., 1998; Wu et al., 2012). It has been shown that crop basal internodes play a key role in resistance to lodging. In particular, the physical strength of basal internodes is fundamental in withstanding self-gravity and external wind (Zhang et al., 2014; Xu et al., 2017). At the same time, basal internode mechanical strength was equally closely related to its chemical composition content, except for its relation to its morphology.

Lignin is a principal constituent of plant secondary cell walls and plays an essential role during plant growth and development (Chen et al., 2011). Lignin deposition can promote stem lignification and improve the mechanical strength of the culm, thereby enhancing the lodging resistance (Yoon et al., 2015). Studies in soybean, wheat, and maize have also shown a significant positive correlation between lignin content of culms and their ability to resist lodging, and a significant negative correlation with the rate of lodging (Zhang et al., 2016b; Sun et al., 2018; LIU et al., 2019). Therefore, it is very necessary to increase culm lignin content to increase culm strength and reduce lodging through suitable agronomic measures in field production. Among the lignin biosynthesis enzymes, the phenylalanine ammonia-lyase (PAL), 4-coumarate:CoA ligase (4CL), cinnamyl alcohol dehydrogenase (CAD), and peroxiredoxins (POD) play crucial roles in the lignin biosynthetic pathway (Boudet et al., 2003). It was earlier revealed that the increase in lignin content of the internode at the maize base was significantly and positively correlated with the PAL, CAD, and POD enzyme activities (Ahmad et al., 2018). The lignin content of soybean and buckwheat stems was also found to be significantly positively correlated with the activity of PAL, 4CL, CAD, and POD enzymes (Wang et al., 2015a; Wen et al., 2020).

Silicon is the second most abundant element of the earth’s crust. Although it is not currently believed to be essential for plants, it has a significant regulatory effect on plant growth (Greger et al., 2018). Leaf spray of silicon solution can upregulate the expression levels of genes involved in lignin biosynthesis in soybean culms to increase lignin accumulation and thus enhance culm strength (Hussain et al., 2021). The silicon fertilizer application could significantly increase rice’s culm strength and grain weight per panicle (Dorairaj et al., 2020). Organic carbon refers to the solid product decomposed by excessive heat (Lehmann and Joseph, 2015). More and more materials from plant bio-waste in rural China are proposed to undergo technical pyrolysis to produce organic carbon fertilizers as a way to reduce the use of chemical nitrogen fertilizers (Pan et al., 2017). When plant wastes were fully utilized, they were used to increase soil fertility and crop yields. It has been reported that the application of organic carbon fertilizer can make up for the obstacle of carbon metabolism caused by insufficient light and improve the photosynthetic characteristics and photosynthetic product content (sucrose and soluble sugar, etc.) of wheat leaves (Lei et al., 2021b). To promote the growth of wheat plants. In addition, spraying organic carbon solution on potato leaves can reduce the malondialdehyde content, weaken the peroxidation of the plasma membrane, and effectively alleviate the autotoxicity of phenolic acid caused by continuous cropping (Shaomeng et al., 2021).

Most of the current research on organic carbon fertilizers is focused on improving soil physio-chemical properties. However, it is still unclear about the effects of organic carbon and silicon fertilizers applied in combination with base fertilizers on rapeseed lodging. Henceforth, in the view of principal importance and the lack of information about the role of organic carbon and silicon fertilizers in the adaptation of plants during lodging stress, we aimed to compare the differences in the effects of two fertilizers on the stem strength of oilseed rapeseed. Therefore, their effect on lignin content, lignin biosynthetic enzymes, and related genes in different developmental stages of two rapeseed varieties was studied.

## Materials and Methods

### Experimental Location and Materials

**FIGURE 1:**
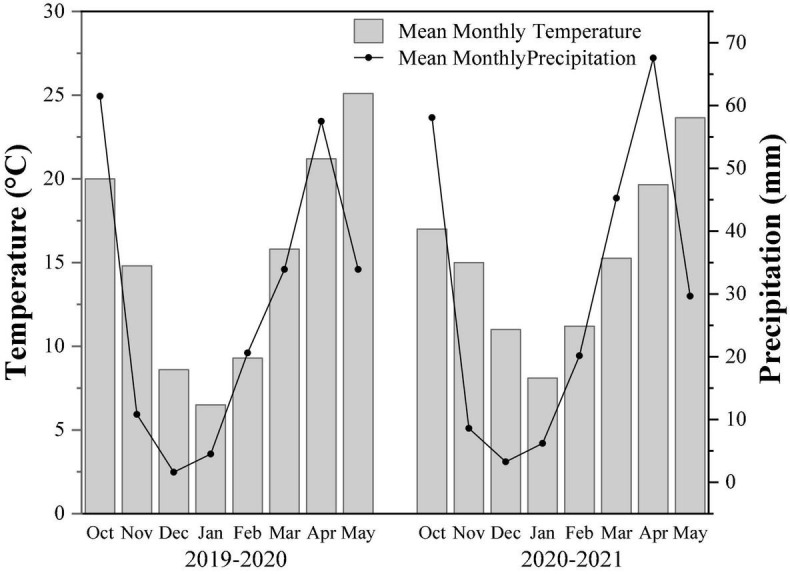
Monthly average temperature and precipitation data for the two-growing season (2019–2021).

The field experiment was conducted at the rapeseed mechanization production demonstration base in Xigao town, Guanghan city, Sichuan province, from October 2019 to May 2021. The area had a humid climate in the middle subtropics. The annual average temperature is 16.3°C, the average precipitation is 890.8 mm, and the average sunshine hours are 1229.2 h. The former crop stubble in the field was rice and alluvial paddy soil at Chengdu Plain. Soil basic fertilities include the pH value 6.05, organic matter 38.80 g/kg, total nitrogen 2.17 g/kg, available phosphorus 16.38 mg/kg, and available potassium 30.8 mg/kg (surface 0–20 cm). The routine meteorological data of the two-year trial are shown in Figure 1.

### Field Experiment Design

The experiments were arranged in a split-plot design with three replications. Each treated plot area was 6 m × 3 m (length × width). Two rapeseed cultivars, namely Jayou and Chuannongyou, with obvious differences in lodging resistance, were selected as experimental materials. Jayou is a lodging-resistant variety, widely cultivated in the upper reaches of the Yangtze River and Chuannongyou is very sensitive to lodging. Four fertilization treatments included: conventional fertilization: NPK compound fertilizer (600 kg/ha) + boron fertilizer (22.5 kg/ha) as control (CK); conventional fertilization + organic carbon fertilizer (300 kg/ha) (CK + OC), conventional fertilization + silicon fertilizer (150 kg/ha) (CK + Si), and conventional fertilization + organic carbon fertilizer (300 kg/ha) + silicon fertilizer (150 kg/ha) (CK + OC + Si) were applied for this experiment. Two varieties were assigned to the main plot and four fertilization treatments to the subplots. Artificial on-demand seeding with a row spacing of 40 cm and hole spacing of 20 cm was used to fix two seedlings per hole after emergence. Silicon fertilizer was purchased from China Yantai Sibaike Fertilizer Co., Ltd. Organic carbon fertilizer was obtained from soybean residue after high-temperature fermentation and purchased from Fujian Oasis Biochemical Co., Ltd. All these fertilizers were applied once as basal fertilizer and urea at 102 kg/ha was used as fertilizer at the sowing stage. Weed and pest control was managed with local conventional management measures.

### Sampling and Measurement

Samples were collected at the bolting, flowering, and pod stages. After the intersegmental collection of the culm base, the samples were put into a liquid nitrogen tank and brought back to the laboratory for cryopreservation for further use.

#### Photosynthetic Rate of Rapeseed Leaves

The net photosynthetic rate was measured by a photosynthesis system (Li-6400XT) equipped with a 6400-02B red/blue LED light source from 10:00 AM to 11:30 AM at 10 days after flowering. The control conditions were manually set to a CO_2_ concentration of 400 μmol CO_2_ mol^–1^, light intensity 1,000 mol m^–2^ s^–1^, sample cell 25°C, leaf temperature (24–26°C), air temperature approximately (25–28°C), and relative humidity of appropriately (65–70%). Three plants were selected to determine the photosynthetic rate of canopy leaves.

#### Plant Agronomic Traits Determinations

At the pod stage, 10 plants with the same growth trend from each treatment were used to determine the agronomic indexes. The plant height was measured with a ruler. The length from the root neck to the stem’s top was considered a plant height. The electronic digital Vernier caliper measured the stem diameter 20 cm above the ground. Internode plumpness was calculated as the ratio of dry weight to internode length.

#### Bending Strength of Stem

The stem was cut 30 cm above the ground and then continued to measure the 10 cm long stem at the cutting position to measure the bending resistance of the stem. Then, the stem was placed in the groove of the stem strength tester (Hangzhou TOP Instrument, China), and continuously applied the pressure to the middle of the stem through a pressure probe until the stem was broken. When the stem was broken, the display screen of the instrument had displayed and recorded the maximum pressure (N) at that time, which was the bending resistance of the stem.

#### Lodging Resistance Index

The culm lodging resistance index was calculated using the following equations:


$$\mathrm{lodging}\,\mathrm{resistance}\,\mathrm{index}=\frac{\mathrm{The}\,\mathrm{bending}\,\mathrm{strength}\,\mathrm{of}\,\mathrm{basal}\,\mathrm{internode}}{\mathrm{The}\,\mathrm{plant}\,\mathrm{height}\times \mathrm{The}\,\mathrm{fresh}\,\mathrm{weight}\,\mathrm{of}\,\mathrm{plant}}\times 100$$

#### Content of Unstructured Carbohydrates

The stems were dried to constant weight at 80°C temperature in the oven. The dried stems were crushed into powder by a micro pulverizer and screened through 60 mesh sieves for standby.15 g of samples were weighed and placed in a 50 ml centrifugal tube. 1.5 ml sulfuric acid (72%) was added to the samples and placed into a shaking water bath pot at 30°C for 1 h. Afterward, 42 ml deionized water was added and then hydrolyzed in a high-temperature sterilizer at 121°C for 2 h. The supernatant of samples was centrifuged and filtered with a.22 μm microporous membrane. The soluble sugar and sucrose contents in the stem were determined by high-performance liquid chromatography (HPLC) (Hussain et al., 2020).

#### Lignin Content and Enzymatic Activities

The lignin contents were measured following the previous method (Klason lignin determination procedure) (Schwanninger and Hinterstoisser, 2002). In brief, 0.3 g of plant dry weight was used as a powder, and 72% of sulfuric acid (H_2_SO_4_) was added to it. After digestion for 1 h, 7.5 ml distilled water was added at 30°C to dilute sulfuric acid concentration to 4%. Afterward, the samples were heated up at 121°C for 1 h. After cooling at room temperature, filtration was done with a single filter paper after drying. Later on, the lignin contents were calculated according to the following formula:


$$\mathrm{Lignin}\,\mathrm{content}=\frac{\mathrm{Residue}\,\mathrm{weight}}{\mathrm{Initial}\,\mathrm{weight}}\times 100\%$$

The lignin enzyme activities (PAL, 4CL, CAD, and POD) were measured using ELISA enzyme-linked immunoassay kits (Solaybo Technology Co., Ltd., Beijing, China).

#### Gene Expression of Lignin Biosynthesis

Fresh stalks were collected 30 cm above the ground and immediately put into liquid nitrogen tanks for storage. Total RNA from the stem was extracted by Trizol kit, and an ultraviolet analyzer determined the RNA concentration after RNA was completely dissolved. RNA was reverse transcribed using RT reagent Kit (Takara Bio Biotechnology Ltd.). The qPCR reaction system was configured concerning Cham QTM Universal SYBR qPCR Master Mix: Vazyme (Nanjing Vazyme Biotech Co., Ltd.) Q71 kit. Using actin as the internal reference gene, the data were analyzed by 2-ΔΔCT methods to determine the relative expression of genes (Livak and Schmittgen, 2001). The transcript sequence was found from the NCBI website, designed with primer 3, primer-blast was used to compare primer specificity, and primer synthesis was carried out by Shanghai Sheng gong Bioengineering Co., Ltd. Three replications of each gene were analyzed. The primer sequences are shown in Table 1.

**TABLE 1:** Primer sequences and amplification products were used in this study.

Gene name	Primer sequence	Products length (bp)
4CL-F	GAATCTAACGGTGCAGGAGGT	158
4CL-R	TCCTCATTCTTAAAATAACCCGTTC	
PAL-F	ATCCCGATATTGTAATGGAGGTT	78
PAL-R	TGATCTCTCCGCCGCATAAC	
C4H-F	GGACAGTTCAGCTTGCACATC	70
C4H-R	AGAAAATTCAAATGGTCCTTGGC	
CAD-F	TGGACTACTCAAACCGAGCG	119
CAD-R	CTCCAATAGCACTTCCCGCA	
POD-F	CACTCCGGTTTAGGAATGGGT	118
POD-R	GCCTTGAGGACCGCTGAATA	
Actin-F	TTTACTCACGAGCTGCTGGC	84
Actin-R	AAGATGATAGACGATTCGAGAGC	

#### Stem Anatomical Structure

The stem was cut off about 20 cm from the ground and quickly put into the test tube containing FAA fixative solution for fixation. After embedding and sectioning, sections were then stained with safranin and solid green staining, dehydrated with ethanol. The sections were 4 μm thick and then stained with 1% safranin solution for 1 h. After decolorizing with alcohol, the sections were stained with a solid green solution for 1 h. After decolorization with ethanol, they were placed under a digital microscope (Nikon 50i, Tokyo, Japan) for observation and photographing.

#### Organic Carbon and Silicon Content Determination of Rapeseed Stem

The organic carbon and silicon contents of the stalks were determined by the elementary TOC analyzer (Almonta Trading Co., Ltd., Shanghai, China) and molybdenum blue methods, respectively (Kraska and Breitenbeck, 2010).

#### Yield

At the maturity stage, the yield of each plot was measured after artificial harvest.

#### Economic Analysis

The economic profit was calculated according to the following formula:

Net income = total income – total cost

Through the oilseed rape Economic Net in China^1^, it can be queried the price of rapeseed acquisition and the prices of compound manure, organic carbon fertilizer, and silicon fertilizer are obtained from the commercial sales of the company in 2019 (the same sales price in 2020).

### Statistical Analysis

For statistical analysis, ANOVA was conducted using SPSS 19. The least significant difference (LSD) was used to examine the differences among treatments of each fertilizer (*P* < 0.05). Pearson’s correlation coefficients were calculated to determine the relationships between the lignin content, activities of lignin-related enzymes, lodging resistance index, and stem bending strength. Tables and graphics were shaped by Excel 2007 and Origin 2017.

## Results

### Effect of Organic Carbon and Silicon Fertilizer on the Photosynthetic Rate of Rapeseed Leaves

**FIGURE 2:**
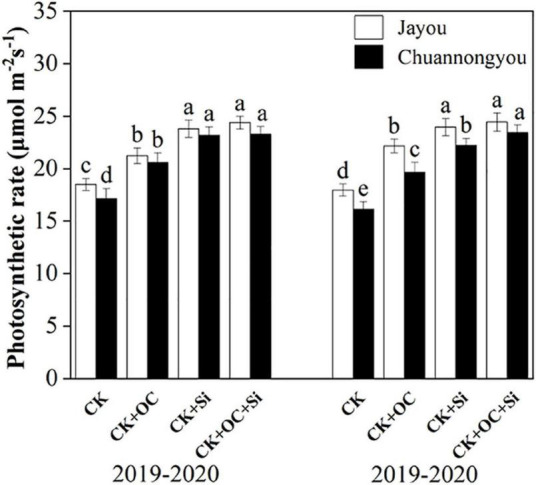
Effect of organic carbon and silicon fertilizer on the photosynthetic rate of rapeseed leaves. Bars represent the means’ standard deviation ± (SD) (*n* = 3). Different lower-case letters indicate significant differences among treatments at *p* ≤ 0.05 (LSD test). The CK, CK + OC, CK + Si, and CK + OC + Si represent the conventional fertilization, conventional + organic carbon fertilization, conventional + silicon fertilization treatment, and conventional + organic carbon + silicon fertilization treatment.

Organic carbon fertilizer and silicon fertilizer significantly increased the photosynthetic rate of rapeseed leaves (Figure 2). In both years, Jayou had a greater photosynthetic rate than Chuannongyou. Jayou exhibited a 6.52% higher photosynthetic rate than Chuannongyou (mean value of all fertilizer treatments). Compared with CK, the photosynthetic rate increased by 20.05, 33.84, and 37.2% under CK + OC, CK + Si, and CK + OC + Si, respectively (the mean value of the two varieties in 2 years), and there was no significant difference between CK + Si and CK + OC + Si.

### Effect of Organic Carbon and Silicon Fertilizer on the Characteristics of Rapeseed Stem

**FIGURE 3:**
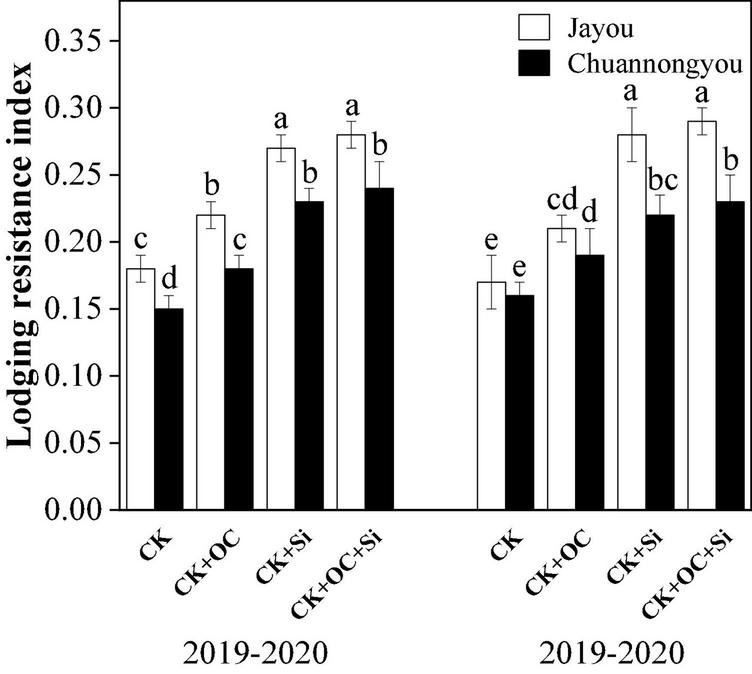
Effect of different fertilization treatments on the lodging resistance index. Different lower-case letters indicate significant differences among treatments at *p* ≤ 0.05 (LSD test). The CK, CK + OC, CK + Si, and CK + OC + Si represent the conventional fertilization, conventional + organic carbon fertilization, conventional + silicon fertilization treatment, and conventional + organic carbon + silicon fertilization treatment.

Different fertilization treatments significantly altered all the studied agronomic variables. All CK + OC, CK + Si, and CK + OC + Si treatments promoted rapeseed plant growth. In contrast to CK, the plant height had significantly enhanced in Jayou and Chuannongyou by 1.49, 3.94, and 4.28% and 2.16, 3.78, and 4.22% under CK + OC, CK + Si, and CK + OC + Si treatment, respectively (based on 2-year averages). The stem diameter of Jayou and Chuannongyou was increased by 15.27, 23.22, and 32.08% and 12.46, 25.48, and 26.83% under CK + OC, CK + Si, and CK + OC + Si, respectively, while the internode plumpness of Jayou and Chuannongyou was augmented by 5.13, 13.99, and 18.03% and 9.19, 15.07, and 24.26% under CK + OC, CK + Si, and CK + OC + Si treatments, respectively. The bending strength of Jayou and Chuannongyou was enlarged by 10.78, 21.72, and 31.45% and 15.73, 29.44, and 31.94% under the CK + OC, CK + Si, and CK + OC + Si (based on 2-year average data), respectively. All of the above-mentioned parameters showed a similar trend in both years under different fertilization treatments (Table 2). The simultaneous addition of organic carbon and silicon fertilizer treatments delayed the onset time of lodging. Moreover, CK + OC and CK + Si treatment significantly improved the lodging resistance index of both varieties, but no significant difference was observed between CK + Si and CK + OC + Si treatment in all the agronomic parameters (Figure 3). Besides this, it was found that the Jayou was superior to Chuannongyou and CK + Si treatment significantly improved all the studied agronomic parameters.

**TABLE 2:** Effects of different fertilizer treatments on stem traits of rapeseed.

Year	varieties	Treatments	Plant height(cm)	Stem diameter(mm)	Internode plumpness	Bending strength(N)	Time of lodging
2019-2020	Jayou	CK	185.67 ± 7.02^c^	13.18 ± 1.03^d^	0.20 ± 0.01^b^	77.67 ± 3.65^cd^	—
		CK + OC	188.89 ± 4.34^bc^	15.44 ± 1.12^c^	0.21 ± 0.01^b^	85.22 ± 4.32^b^	—
		CK + Si	191.88 ± 6.51^abc^	17.59 ± 0.96^a^	0.23 ± 0.03^a^	101.33 ± 6.06^a^	—
		CK + OC + Si	192.35 ± 6.75^abc^	17.61 ± 0.54^a^	0.23 ± 0.02^a^	102.12 ± 4.12^a^	—
	Chuannongyou	CK	188.00 ± 5.73^c^	12.36 ± 1.21^d^	0.16 ± 0.01^d^	62.37 ± 3.15^e^	FS3.18
		CK + OC	192.67 ± 7.37^abc^	14.58 ± 1.83^c^	0.18 ± 0.02^c^	74.67 ± 4.08^d^	FS3.23
		CK + Si	195.11 ± 4.44^ab^	16.40 ± 1.22^b^	0.20 ± 0.01^b^	81.33 ± 4.62^bc^	PS4.10
		CK + OC + Si	196.35 ± 5.65^a^	16.41 ± 0.84^b^	0.21 ± 0.02^b^	84.26 ± 3.62^bc^	PS4.12
2020-2021	Jayou	CK	184.13 ± 4.21^e^	13.06 ± 0.85^cd^	0.19 ± 0.02^cd^	74.42 ± 4.26^c^	—
		CK + OC	186.41 ± 4.64^d^	14.81 ± 1.22^b^	0.20 ± 0.01^cd^	83.23 ± 5.06^b^	—
		CK + Si	192.46 ± 5.03^bc^	16.95 ± 1.47^a^	0.22 ± 0.02^ab^	95.67 ± 3.66^a^	—
		CK + OC + Si	193.24 ± 6.06^ab^	17.05 ± 1.21^a^	0.23 ± 0.01^a^	97.80 ± 5.37^a^	—
	Chuannongyou	CK	187.48 ± 6.12^d^	12.64 ± 1.36^d^	0.17 ± 0.01^e^	64.45 ± 4.85^d^	FS3.22
		CK + OC	190.93 ± 4.07^c^	13.52 ± 0.78^c^	0.18 ± 0.01^de^	72.01 ± 4.68^c^	FS3.27
		CK + Si	194.58 ± 3.68^a^	14.95 ± 1.37^b^	0.20 ± 0.02^bc^	82.81 ± 3.88^b^	PS4.50
		CK + OC + Si	194.96 ± 4.44^a^	15.28 ± 0.93^b^	0.21 ± 0.01^bc^	83.00 ± 4.42^b^	PS4.60
		V	*	*	*	ns	*
		T	ns	*	ns	ns	**
		V*T	ns	ns	ns	ns	ns

Different lower-case letters indicate significant differences among treatments at p ≤ 0.05 (LSD test) with the means (n = 3) of values. The CK, CK + OC, CK + Si, and CK + OC + Si represent the conventional fertilization, conventional + organic carbon fertilization, conventional + silicon fertilization treatment, and conventional + organic carbon + silicon fertilization treatment. V, T, and Y represent the variety, treatment, and year, FS and PS showed the flowering and pod stages while * indicates the significance, respectively. ** means “highly significant”.

### Effect of Organic Carbon and Silicon Fertilizer on Carbohydrates of Rapeseed Stems

The changing trend of the carbohydrates content of the two varieties was the same in both years. The organic carbon and silicon fertilizer increased culm sucrose, soluble sugar, and starch contents. There was no significant difference in sucrose content between the two cultivars under CK treatment, but the difference reached up to significant level under the other fertilization treatments. In Jayou and Chuannongyou, the maximum value was reached under CK + Si and CK + OC + Si treatment, 69.09 and 55.32% and 69.33 and 57.11% higher than CK, respectively. The Jayou showed significantly higher sucrose content than Chuannongyou under CK + OC and CK + Si treatments.

The soluble sugar content of Jayou and Chuannongyou showed the following trend under various fertilization treatments: CK + OC + Si > CK + Si > CK + OC > CK in both years. Compared with CK, the soluble sugar contents of Jayou and Chuannongyou under CK + OC, CK + Si, and CK + OC + Si were enhanced by 25.52, 111.88, and 113.93% and 57.98, 117.78, and 120.63%, respectively (average value based on 2 years). The soluble sugar content of Chuannongyou was significantly higher than that of Jayou under all the treatments. Moreover, the starch content of both genotypes showed the following trend: CK + OC + Si > CK + Si > CK + OC > CK. Contrary to CK, the starch content of Jayou was significantly improved by 21.59, 53.17, and 54.95% under the application of CK + OC, CK + Si, and CK + OC + Si, respectively, while starch content of Chuannongyou was remarkably increased by 39.03, 119.09, and 119.61% under CK + OC, CK + Si, and CK + OC + Si, respectively (mean value based on two years). Furthermore, the starch content of Chuannongyou showed a significant increase as compared to Jayou (Table 3). Moreover, the carbohydrate content of both genotypes was substantially increased by CK + Si treatment compared to other fertilizations.

**TABLE 3:** Effects of different fertilizer treatments on non-structural carbohydrates.

Year	Varieties	Treatments	Sucrose (mg/g)	Soluble sugar (mg/g)	Starch (mg/g)
2019–2020	Jayou	CK	8.22 ± 0.56^d^	2.73 ± 0.24^d^	5.35 ± 0.34^d^
		CK + OC	10.87 ± 0.67^b^	3.28 ± 0.19^c^	6.32 ± 0.32^c^
		CK + Si	14.14 ± 0.72^a^	5.55 ± 0.32^a^	7.70 ± 0.47^b^
		CK + OC + Si	14.18 ± 0.41^a^	5.62 ± 0.42^a^	7.78 ± 0.38^b^
	Chuannongyou	CK	7.43 ± 0.68^e^	2.44 ± 0.18^e^	3.52 ± 0.22^f^
		CK + OC	8.99 ± 0.55^dc^	3.96 ± 0.27^b^	5.08 ± 0.29^e^
		CK + Si	10.72 ± 0.41^b^	5.46 ± 0.36^a^	8.23 ± 0.42^a^
		CK + OC + Si	10.84 ± 0.51^b^	5.48 ± 0.24^a^	8.25 ± 0.37^a^
2020–2021	Jayou	CK	7.86 ± 0.25^d^	2.59 ± 0.42^f^	4.87 ± 0.42^e^
		CK + OC	9.68 ± 0.51^c^	3.39 ± 0.36^e^	6.09 ± 0.28^c^
		CK + Si	12.98 ± 0.44^a^	5.71 ± 0.51^a^	7.91 ± 0.33^b^
		CK + OC + Si	13.06 ± 0.42^a^	5.75 ± 0.47^a^	8.01 ± 0.27^b^
	Chuannongyou	CK	6.63 ± 0.38^e^	2.46 ± 0.37^g^	4.12 ± 0.51^f^
		CK + OC	8.25 ± 0.62^d^	3.78 ± 0.46^d^	5.51 ± 0.37^d^
		CK + Si	11.03 ± 0.38^b^	5.21 ± 0.28^c^	8.42 ± 0.43^a^
		CK + OC + Si	11.16 ± 0.44^b^	5.33 ± 0.48^b^	8.44 ± 0.34^a^
		V*T	*	ns	*
		Y	ns	ns	ns

Different lower-case letters indicate significant differences among treatments at p ≤ 0.05 (LSD test) with the means (n = 3) of values. The CK, CK + OC, CK + Si, and CK + OC + Si represent the conventional fertilization, conventional + organic carbon fertilization, conventional + silicon fertilization treatment, and conventional + organic carbon + silicon fertilization treatment. V, T, and Y represent the variety, treatment, and year, while * and ns indicate the significance and non-significance, respectively.

### Effect of Organic Carbon and Silicon Fertilizer on Lignin Content of Oilseed Rapeseed Basal Internode

Compared with CK, both CK + OC and CK + Si treatments significantly increased shoot lignin content, and the maximum values were observed in the CK + OC + Si treatment at all three stages. Still, no significant difference was observed between CK + Si and CK + OC + Si treatments. In Jayou, the lignin content was enhanced by 18.06, 42.38, and 44.87% under CK + OC, CK + Si, and CK + OC + Si, respectively (mean based on 2 years and three stages). The mean value, obtained at three stages and 2 years of Chuannongyou, displayed an increase of 18.55, 59.05, and 60.58% under the application of CK + OC, CK + Si, and CK + OC + Si, respectively. In comparing both varieties, Chuannongyou showed a higher value of lignin content under different fertilization in both years (Table 4). Compared to other fertilizations, CK + Si treatment considerably increased the lignin concentration in both genotypes.

**TABLE 4:** Effects of different fertilizer treatments on lignin content of rapeseed stem.

Year	Varieties	Treatments	Lignin content(mg/g)		
			
			Bolting stage	Flowering stage	Pod stage
2019–2020	Jayou	CK	43.41 ± 1.02^c^	66.84 ± 1.42^c^	77.20 ± 0.91^c^
		CK + OC	46.42 ± 0.96^b^	82.32 ± 0.92^b^	93.76 ± 1.41^b^
		CK + Si	58.39 ± 0.75^a^	93.72 ± 1.30^a^	114.32 ± 1.54^a^
		CK + OC + Si	60.02 ± 0.62^a^	95.03 ± 0.75^a^	116.26 ± 0.96^a^
	Chuannongyou	CK	31.33 ± 0.86^e^	43.72 ± 1.05^e^	56.56 ± 1.22^d^
		CK + OC	36.70 ± 1.17^d^	52.56 ± 0.82^d^	61.43 ± 0.88^d^
		CK + Si	43.71 ± 0.97^c^	76.31 ± 1.17^b^	82.66 ± 0.97^c^
		CK + OC + Si	44.72 ± 0.52^c^	77.26 ± 0.85^b^	83.52 ± 1.05^c^
2020–2021	Jayou	CK	41.43 ± 0.75^e^	64.67 ± 0.58^d^	79.03 ± 0.71^d^
		CK + OC	48.07 ± 0.81^c^	80.28 ± 0.85^b^	92.30 ± 1.21^b^
		CK + Si	59.43 ± 0.84^b^	94.63 ± 1.12^a^	111.93 ± 0.82^a^
		CK + OC + Si	61.27 ± 0.77^a^	95.60 ± 0.97^a^	112.57 ± 1.34^a^
	Chuannongyou	CK	29.03 ± 0.68^g^	41.08 ± 0.75^f^	54.83 ± 0.66^f^
		CK + OC	35.05 ± 1.04^f^	53.43 ± 1.41^e^	63.17 ± 0.72^e^
		CK + Si	45.32 ± 0.93^d^	78.25 ± 0.82^c^	80.86 ± 1.08^c^
		CK + OC + Si	45.33 ± 0.69^d^	78.77 ± 0.68^c^	81.40 ± 0.87^c^
		V*T	*	*	*
		Y	ns	ns	ns

Different lower-case letters indicate significant differences among treatments at p ≤ 0.05 (LSD test) with the means (n = 3) of values. The CK, CK + OC, CK + Si, and CK + OC + Si represent the conventional fertilization, conventional + organic carbon fertilization, conventional + silicon fertilization treatment, and conventional + organic carbon + silicon fertilization treatment. V, T, and Y represent the variety, treatment, and year, while * and ns indicate the significance and non-significance, respectively.

### Lignin Biosynthesis Enzyme Activities in Rapeseed Stem

**FIGURE 4:**
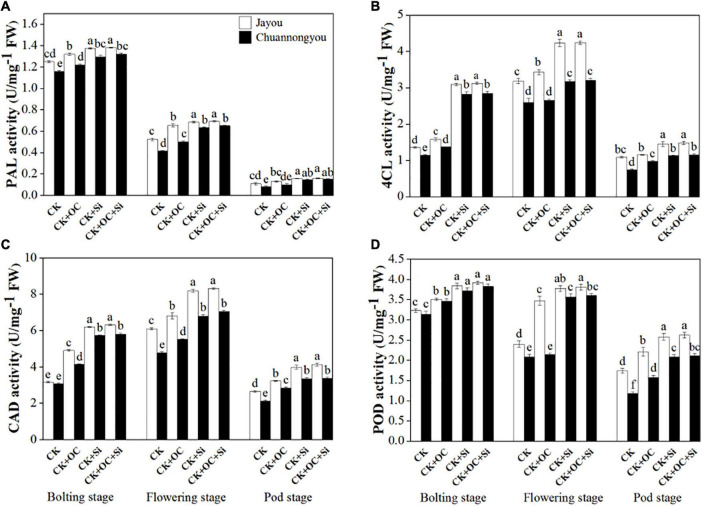
Effect of organic carbon and silicon fertilizers on the enzymes related to lignin biosynthesis in rapeseed stems **(A)** PAL, phenylalanine ammonia-lyase activity, **(B)** 4CL, 4-coumarate: CoA ligase activity, **(C)** CAD, cinnamyl alcohol dehydrogenase activity, and **(D)** POD, peroxiredoxins activity. Bars represent the means ± *SD* (*n* = 3). Different lower-case letters indicate significant differences among treatments at *p* ≤ 0.05 (LSD test). The CK, CK + OC, CK + Si, and CK + OC + Si represent the conventional fertilization, conventional + organic carbon fertilization, conventional + silicon fertilization treatment, and conventional + organic carbon + silicon fertilization treatment.

Our results about enzymatic activities unveiled the significant impact of different fertilizers on the lodging resistance capacity of brassica cultivars at different developmental stages. According to our findings, the PAL activity gradually declined from the bolting to pod stage (Figure 4A). As compared to CK, CK + OC, CK + Si, and CK + OC + Si improved the PAL activity of Jayou by 16.76, 28.66 and 30.39%, respectively, and Chuannongyou showed an increase of 16.12, 47.3, and 51.54% under CK + OC, CK + Si, and CK + OC + Si fertilization, respectively (means based on three growth stages) (Figure 4A). At all three developmental stages, the PAL activity showed no significant difference under CK + Si and CK + OC + Si treatment.

Both 4CL and CAD activities exhibited unimodal variation curves (Figures 4B,C), with each treatment peaking at the flowering stage. An increment of 9.85, 64.42, and 66.17% was observed in the 4CL activity of Jayou under CK + OC, CK + Si, and CK + OC + Si when compared with CK, respectively. However, the 4CL activity of Chuannongyou was augmented by 17.99, 74.45, and 76.32% under the treatment of CK + OC, CK + Si, and CK + OC + Si, respectively (average values based on three growth stages) (Figure 4B). The exogenous application of CK + OC, CK + Si, and CK + OC + Si improved the CAD activity of both cultivars. The mean results obtained at three growth stages showed that CK + OC, CK + Si, and CK + OC + Si treatments increased the CAD activity of Jayou by 29.55, 60.17, and 63.85%, respectively. While, the CAD activity of Chuannongyou was enhanced by 28.12, 62.34, and 64.97% under CK + OC, CK + Si, and CK + OC + Si, respectively (Figure 4C). Likewise, the POD activity was also improved by the exogenous fertilization of organic carbon and silicon at all the studied developmental stages of brassica cultivars. Compared with CK, the POD activity of Jayou at three stages average value displayed an increase of 26.75, 41.55, and 43.56% under CK + OC, CK + Si, and CK + OC + Si, respectively. The CK + OC, CK + Si, and CK + OC + Si treatments significantly increased the POD activity of Chuannongyou by 15.38, 55.46, and 57.96%, respectively, compared with CK treatment (Figure 4D). In a comparison of both varieties, our findings revealed that Chuannongyou cultivar exhibited higher and more significant enzymatic activities than Jayou under CK + Si fertilization. Based on our results, CK + Si fertilization is a viable method for increasing rapeseed enzymatic activity.

### Expression of Lignin Biosynthesis Key Genes in Rapeseed Stem

**FIGURE 5:**
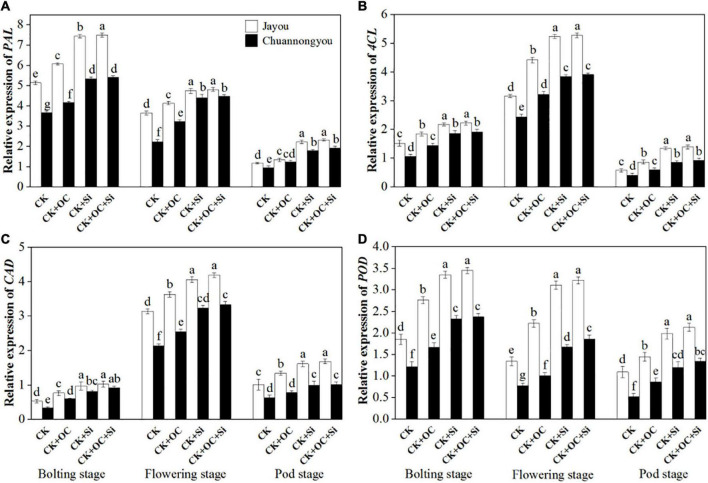
Effect of organic carbon and silicon fertilizers on the expression of genes related to lignin biosynthesis in rapeseed stems **(A)** PAL, phenylalanine ammonia-lyase activity, **(B)** 4CL, 4-coumarate: CoA ligase activity, **(C)** CAD, cinnamyl alcohol dehydrogenase activity, and **(D)** POD, peroxiredoxins activity. Bars represent mean ± *SD* (*n* = 3). Different lower-case letters indicate significant differences among treatments at *p* ≤ 0.05 (LSD test). The CK, CK + OC, CK + Si, and CK + OC + Si represent the conventional fertilization, conventional + organic carbon fertilization, conventional + silicon fertilization treatment, and conventional + organic carbon + silicon fertilization treatment.

All the investigated genes in this study are the key determinants of lodging resistance in plants. The gene expression analysis of the investigated genes showed a similar trend with enzymatic activities. The *PAL* gene expression displayed a gradual decline from the bolting to pod stage (Figure 5A). Compared to CK, CK + OC, CK + Si, and CK + OC + Si improved the *PAL* gene expression of Jayou by 15.48, 55.09, and 58.35%, respectively. Whereas Chuannongyou showed an increase of 29.04, 74.67, and 83.14% under CK + OC, CK + Si, and CK + OC + Si fertilization, respectively (means based on three growth stages) (Figure 5A). It is further observed that, at all the investigated developmental stages, the *PAL* upregulation was greater under CK + OC + Si treatment as compared to CK and CK + OC and CK + Si.

The transcriptional levels of *4CL* and *CAD* genes also exhibited the unimodal variation curves (Figures 4B,C), with each treatment peaking at the flowering stage. Contrary to the control, the mean value of three stages showed that the 4CL transcriptional level of Jayou was upregulated by 37.87, 81.53, and 86.35% under CK + OC, CK + Si, and CK + OC + Si fertilization, respectively. However, the CK + OC, CK + Si, and CK + OC + Si enhanced the *4CL* gene expression of Chuannongyou by 39.01, 82.76, and 90.94%, respectively (Figure 5B). Furthermore, contrary to CK, an increment of 29.86, 56.77, and 63.45% was detected in the transcription of the *CAD* gene of Jayou cultivar when the plants were treated with CK + OC, CK + Si, and CK + OC + Si fertilization, respectively. While the *CAD* gene expression of Chuannongyou was enhanced by 41.61, 83.56, and 98.48% under CK + OC, CK + Si, and CK + OC + Si, respectively (mean based on three stages) (Figure 5C).

In our experimentation, the application of CK + OC, CK + Si, and CK + OC + Si had upregulated the *POD* gene expression of Jayou by 49.34, 98.55, and 107.71%, respectively (mean value at three stages). Compared with CK, the *POD* transcriptional level of Chuannongyou displayed an increase of 43.98, 112.27, and 130.98% under CK + OC, CK + Si, and CK + OC + Si, respectively (Figure 5D). Overall, we found that Chuannongyou showed improved lodging resistance under CK + Si and CK + OC + Si fertilization compared to Jayou, which is accompanied by the higher upregulated of studied genes. Moreover, under CK + OC treatment, the response of both varieties is not specific. In both genotypes, CK + Si treatment significantly increased gene expression and enhanced the lodging resistance index compared to other fertilizations.

### Anatomical Observation on Stem Tissue of Rapeseed

**FIGURE 6:**
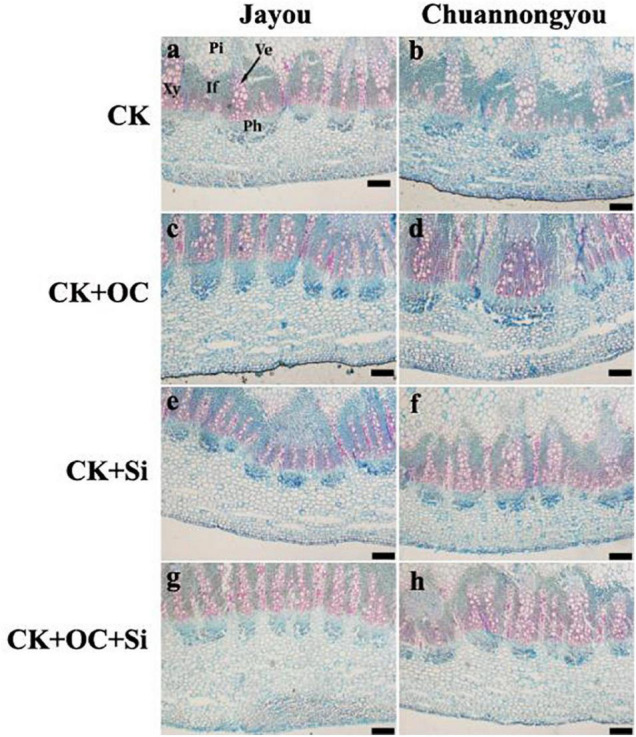
Reflection of stem anatomical structure. Where, If, interfascicular; Ph, phloem; Pi, pith; Xy, xylem; Ve, vessel. Bar = 200 mm. The CK **(A,B)**, CK + OC **(C,D)**, CK + Si **(E,F)**, and CK + OC + Si **(G,H)** represent the conventional fertilization, conventional + organic carbon fertilization, conventional + silicon fertilization treatment, and conventional + organic carbon + silicon fertilization treatment.

Safranin fast green staining was applied to the culm sections to observe the anatomical structure of the rapeseed stem. A thorough investigation observed that lignin was stained red, and the darker the color, the more lignin deposition. The redder shade was found in Jayou than Chuannongyou, indicating that Jayou had deposited more lignin content. Compared with the control, different fertilization treatments increased the deposition of lignin around the thick wall tissue cells and vascular bundle sheath, which was the reason for the much darker color in stem tissue (Figure 6).

Different fertilizer treatments had no significant effect on the epidermal thickness of the two varieties. The maximum values of cortical thickness, vascular bundle area, mechanical structure layer thickness, xylem area, and pith area were observed under CK + OC + Si treatment, which increased by 9.49, 34.32, 11.04, 52.37, and 30.56%, respectively, as compared with CK (based on the average of two variations). However, there was no significant difference between CK + Si and CK + OC + Si treatment (Table 5). Additionally, both cultivars benefited greatly from the treatment of CK + Si in terms of epidermal thickness and xylem area.

**TABLE 5:** Effect of different fertilization treatment on stem tissue of rapeseed.

Varieties	Treatment	Epidermis thickness (μm)	Cortical thickness (μm)	Vascular bundle area (mm^2^)	Mechanical structure thickness(μm)	Xylem area (mm^2^)	Pith area (mm^2^)
Jayou	CK	16.29 ± 0.68^a^	473.70 ± 6.60^cd^	22.00 ± 0.86^d^	554.24 ± 8.04^c^	16.17 ± 0.89^c^	112.60 ± 4.95^d^
	CK + OC	16.54 ± 0.86^a^	503.07 ± 8.16^b^	25.27 ± 0.82^b^	580.93 ± 12.44^b^	19.13 ± 0.52^b^	126.90 ± 6.76^bc^
	CK + Si	16.68 ± 0.83^a^	518.97 ± 6.68^a^	28.13 ± 0.88^a^	587.90 ± 8.56^b^	23.00 ± 0.94^a^	131.07 ± 6.44^b^
	CK + OC + Si	16.98 ± 0.85^a^	525.50 ± 5.09^a^	29.10 ± 0.72^a^	611.43 ± 9.07^a^	23.81 ± 0.25^a^	142.47 ± 11.36^a^
Chuannongyou	CK	14.42 ± 0.57^b^	461.37 ± 8.52^d^	17.43 ± 0.85^f^	401.03 ± 10.45^f^	9.08 ± 0.50*^f^*	96.83 ± 4.90^e^
	CK + OC	14.76 ± 0.97^b^	471.70 ± 6.35^cd^	19.37 ± 0.81^e^	426.57 ± 15.50^e^	11.75 ± 0.23^e^	108.63 ± 4.55^d^
	CK + Si	14.75 ± 0.56^b^	482.77 ± 7.74^c^	22.73 ± 0.90^cd^	439.70 ± 8.41^d^	14.15 ± 0.55^d^	122.30 ± 2.72^c^
	CK + OC + Si	14.90 ± 0.99^b^	497.40 ± 8.16^c^	23.77 ± 0.83^bc^	448.23 ± 4.24^d^	14.30 ± 0.62^d^	130.33 ± 4.74^b^
	V	*	*	*	*	*	*
	T	ns	*	*	*	*	*
	V*T	ns	*	ns	ns	*	ns

Different lower-case letters indicate significant differences among treatments at p ≤ 0.05 (LSD test) with the means (n = 3) of values. The CK, CK + OC, CK + Si, and CK + OC + Si represent the conventional fertilization, conventional + organic carbon fertilization, conventional + silicon fertilization treatment, and conventional + organic carbon + silicon fertilization treatment. V, T, and Y represent the variety, treatment, and year, while * and ns indicate the significance and non-significance, respectively.

### Organic Carbon and Silicon Content Determination of Rapeseed Stem

**FIGURE 7:**
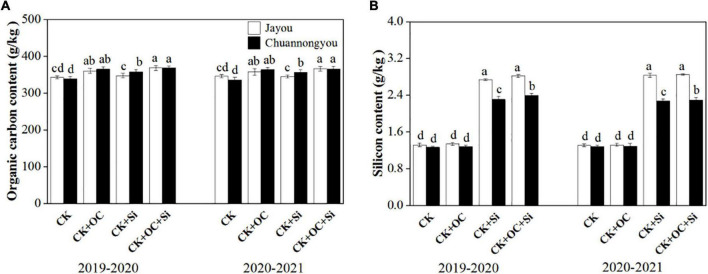
Effect of different fertilization on organic stem carbon **(A)** and silicon content **(B)** of rapeseed. Different lower-case letters indicate significant differences among treatments at *p* < 0.05 (LSD test) with the means (*n* = 3) of values. The CK, CK + OC, CK + Si, and CK + OC + Si represent the conventional fertilization, conventional + organiccarbon fertilization, conventional + silicon fertilization treatment, and conventional + organic carbon + silicon fertilization treatment.

Compared with CK, the organic stem carbon and silicon content of Jayou and Chuannongyou increase by 8.45 and 4.18% and 8.88 and 3.72%, 3.43 and 83.51% and 3.50 and 78.71% under CK + OC and CK + Si treatments, respectively (Based on the two-year mean values) (Figure 7). The maximum stem organic carbon content and silicon content values appeared under the CK + OC and CK + OC + Si treatments, but CK + Si significantly showed higher silicon content in both varieties. Additionally, Jayou stem showed higher values of silicon content in both years.

### Effect of Organic Carbon and Silicon Fertilizer on Yield

We found that CK + OC did not significantly affect branch number, siliques number, and 1,000 grain weight (Table 6). However, the CK + Si treatment significantly improved the number of pods and yield by 2.17 and 20.91%, 0.85 and 10.37% in Jayou and Chuannongyou, respectively while compared with CK (based on average values of 2 years data). Furthermore, there was no significant difference between the number of pods and yield between CK + Si and CK + OC + Si treatments. Compared to other fertilization, the application of CK + Si significantly improved the yield parameter.

**TABLE 6:** Effects of different fertilizer treatments on yield components.

	Treatments	Number of branches	Number of pods/plant	Number of seeds/pod	1000 grain weight(g)	Yield (kg/ha)
Jayou	CK	5.25 ± 1.25^c^	270.51 ± 20.5^b^	22.25 ± 1.02^bc^	4.09 ± 0.15^a^	3633.02 ± 105.33^c^
	CK + OC	5.00 ± 2.01^c^	275.33 ± 31.25^b^	22.50 ± 0.85^b^	4.05 ± 0.08^a^	3680.67 ± 91.56^c^
	CK + Si	5.75 ± 1.34^bc^	311.29 ± 31.25^a^	24.50 ± 0.52^a^	4.07 ± 0.13^a^	3953.00 ± 122.71^a^
	CK + OC + Si	6.00 ± 1.06^abc^	313.54 ± 25.11^a^	24.55 ± 1.21^a^	4.04 ± 0.14^a^	3960.11 ± 105.33^a^
Chuannongyou	CK	7.05 ± 1.32^a^	204.01 ± 33.65^e^	20.53 ± 1.27^c^	4.03 ± 0.07^a^	3389.56 ± 144.11^d^
	CK + OC	6.75 ± 1.52^ab^	212.25 ± 20.68^d^	21.52 ± 0.85^bc^	4.05 ± 0.11^a^	3480.00 ± 116.25^d^
	CK + Si	5.76 ± 1.38^bc^	258.65 ± 38.15^c^	22.50 ± 1.12^b^	4.11 ± 0.16^a^	3786.33 ± 85.62^b^
	CK + OC + Si	7.12 ± 1.08^a^	264.15 ± 19.26^c^	22.52 ± 0.82^b^	4.07 ± 0.08^a^	3812.67 ± 88.86^b^
	V	*	*	*	ns	*
	T	ns	*	ns	ns	**
	V*T	ns	ns	ns	ns	ns

Different lower-case letters indicate significant differences among treatments at p ≤ 0.05 (LSD test) with the means (n = 3) of values. The CK, CK + OC, CK + Si, and CK + OC + Si represent the conventional fertilization, conventional + organic carbon fertilization, conventional + silicon fertilization treatment, and conventional + organic carbon + silicon fertilization treatment. V, T, and Y represent the variety, treatment, and year, while * and ns indicate the significance and non-significance, respectively. ** means “highly significant”.

### Economic Analysis

The addition of both organic carbon and silicon fertilizer treatments had enhanced the costs and net income (Table 7). It can be seen that the CK + OC and CK + OC + Si treatment had a higher cost than the CK + Si treatment, which was associated with a 35.4 and 20.25% increase in total CK costs, respectively. Compared with CK, CK + Si treatments increased net income by 7.32%, while CK + OC treatment decreased the net income by 12.49% (based on two varieties means). Thus, CK + Si treatment considerably boosted rapeseed economic profit of both varieties.

**TABLE 7:** Effect of different fertilization treatments on cost and income.

		Fertilizer						
		
Varieties	Treatment	Compound manure (kg ha^–1^)	Boron fertilizer (kg ha^–1^)	Organic C fertilizer (kg ha^–1^)	Silicon fertilizer (kg ha^–1^)	Total costs (RNB)	Yield (kg)	Net income (RNB)
Jayou	CK	600	22.5	—	—	2962.50d	3633.02bc	7936.56b
	CK + OC	600	22.5	750	—	4012.50b	3680.67b	7029.51cd
	CK + Si	600	22.5	—	150	3562.50c	3953.00a	8296.50a
	CK + OC + Si	600	22.5	750	150	4612.50a	3960.11a	7267.83c
Chuannongyou	CK	600	22.5	—	—	2962.50d	3389.56d	7206.18b
	CK + OC	600	22.5	750	—	4012.50b	3480.00c	6427.50d
	CK + Si	600	22.5	—	150	3562.50c	3786.33ab	7796.49a
	CK + OC + Si	600	22.5	750	150	4612.50a	3812.67a	6825.51c

*Different lower-case letters indicate significant differences among treatments at p ≤ 0.05 (LSD test) with the means (n = 3) of values. The CK, CK + OC, CK + Si, and CK + OC + Si represent the conventional fertilization, conventional + organic carbon fertilization, conventional + silicon fertilization treatment, and conventional + organic carbon + silicon fertilization treatment.*

### Correlation Analysis

**FIGURE 8:**
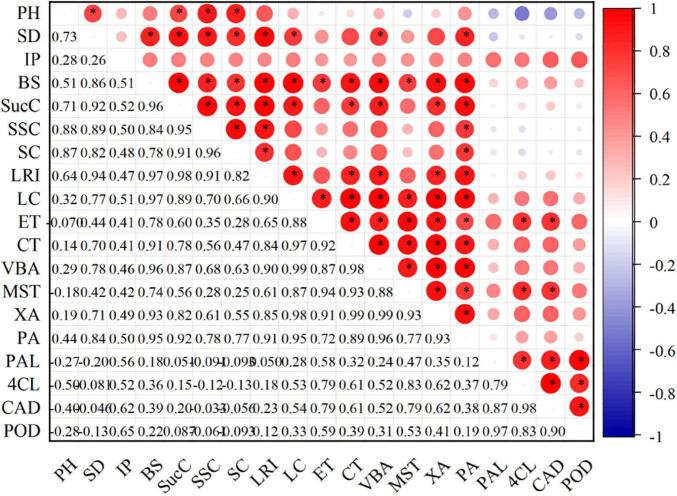
Relationship between stem characteristics, lignin content, and related enzyme activities. Red represents positive correlation, and blue represents negative correlation (*p ≤ 0.05). The intensity of color represents the significance of a variable. PH, plant height; SD, stem diameter; IP, internode plumpness; BS, bending strength; SucC, sucrose content; SSC, soluble sugar content; SC, starch content; LRI, lodging resistance index; LC, lignin content; ET, epidermis thickness; CT, cortical thickness; VBA, vascular bundle area; MST, mechanical structure thickness; XA, xylem area; PA, pith area. PAL, phenylalanine ammonia-lyase activity; 4CL, 4-coumarate: CoA ligase activity; CAD, cinnamyl alcohol dehydrogenase activity; POD, peroxiredoxins activity.

The correlation analysis of the current study had revealed that the stem bending, stem diameter, internode fullness, and lodging resistance index were significantly correlated with lignin content. It is further found that the PAL, 4CL, CAD, and POD enzyme activities were also significantly correlated with lignin content. These observations showed that lignin metabolism was closely related to stem lodging resistance. Moreover, the organic carbon and silicon contents of the stem were positively correlated with stem diameter, bending strength, lodging resistance index, and lignin content. Still, a significant correlation was observed for silicon content (Figure 8).

## Discussion

### Plant Stem Characteristics

At present, there are few studies on the effect of organic carbon and silicon fertilizers on plant stem morphology and bending strength. However, the present study is tempted to evaluate the impact of various fertilizer treatments on rapeseed culm morphology, lignin accumulation, and their relationships with their lodging resistance capacities. Stalk morphology is closely related to lodging (Rajkumara, 2008; Berry and Berry, 2015). Under the treatment of CK + OC + Si, the plant morphological development was the best, but there was no significant difference with CK + Si. In the current study, both organic carbon and silicon fertilizers improved plant height, stem diameter, internode plumpness, and stalk strength (Table 2). Stem diameter and internode plumpness are closely related to stem lodging resistance. The higher the internode plumpness, the more stem dry matter accumulation, tighter tissue, and stronger compression resistance (Xue et al., 2016). It is generally considered that too high plant height is unfavorable to crop lodging because the increase of plant height will lead to the upward shift of plant center of gravity and aggravate the risk of lodging (Salman et al., 2012; Caterina et al., 2017). Although the plant height in CK + Si treatment was higher than that in CK + OC treatment, the CK + Si treatment had higher stem bending strength. Therefore, CK + Si has stronger lodging resistance and a higher lodging resistance coefficient. Stalk strength is often more critical, especially the mechanical strength of stalk base plays a key role in bearing plant weight and external pressure (Zhang et al., 2016b; Zheng et al., 2017). Stalk bending strength is an essential mechanical index of stem strength, which is significantly negatively correlated with field lodging rate (Xue et al., 2016; Kamran et al., 2018a). Our results showed that both carbon-silicon fertilizers promoted rapeseed plant growth and improved stem quality. There is a significant positive correlation between bending strength and lodging resistance index, Jayou’s stalk bending strength is significantly higher than Chuannongyou, so it has stronger lodging resistance. The bending strength of stem under silicon fertilizer was markedly higher than that under organic carbon fertilizer. This may be related to the fact that silicon can enhance the strength and stiffness of cell walls during stem deposition (Ma and Yamaji, 2006). It was also examined that silicon could promote cell silicification and improve cell wall structure integrity (Lei et al., 2021a). The previous studies showed that the application of silicon improved the stem strength and silicon content of rice and soybean (Liang et al., 2013; Hussain et al., 2021).

### Stem Lignin Metabolism

Lodging is an important factor leading to reducing crop yield and quality (Okuno et al., 2014). Lignin is a typical phenolic compound, which is sensitive to light. Lignin is mainly regulated by sucrose, light, and biological circadian rhythm (Zhao and Dixon, 2011). Lignin fills the secondary cell wall to give the stem mechanical strength (Ma, 2010). Many studies have shown that lignin content is closely related to crop stem strength. Increasing stem lignin content can improve stalk strength. The results reported in this paper are consistent with the previous research results. The lignin content of rapeseed stalk increased gradually during growth and was significantly positively correlated with stalk bending strength and lodging resistance index. The results showed that lignin played an important role in the formation of stalk strength (Jo Heuschele et al., 2020). The difference in the effect of organic carbon fertilizer and silicon fertilizer on stem lignin content may be related to the accumulation of carbohydrates. Carbohydrates are the basis for the formation of stem mechanical strength. It was found that the increase of lignin content in the stem is often accompanied by the increase of non-structural carbohydrates in stems, such as sucrose, starch, and other carbohydrates (Wu et al., 2017; Wen et al., 2020). Sucrose is the carbon source of lignin synthesis (Rogers et al., 2005). The cane sucrose content is higher under silicon fertilizer treatment, providing more carbon sources for lignin synthesis. Different photosynthetic performances may cause this difference. The photosynthetic rate of leaves treated with silicon fertilizer was significantly higher than that treated with organic carbon fertilizer, which also reflected this (Figure 2). It was reported that silicon increases leaf chlorophyll content, expands chloroplast, and upregulated genes related to light-harvesting complex II, which increases the photosynthetic rate (Teixeira et al., 2020). This means that light energy is a critical environmental component impacting the metabolism, synthesis, and transport of photosynthetic products in crops. It also has a considerable impact on lignin synthesis (Wang et al., 2012). Organic carbon fertilizer can also improve photosynthetic performance, but the accumulation of photosynthetic products is less than that of silicon fertilizer. The increase of photosynthetic rate increased non-structural carbohydrate content (Zhang et al., 2018). Therefore, the leaves treated with silicon fertilizer as the “source” can export more carbohydrates to non-photosynthetic tissues (Rolland et al., 2001).

**FIGURE 9:**
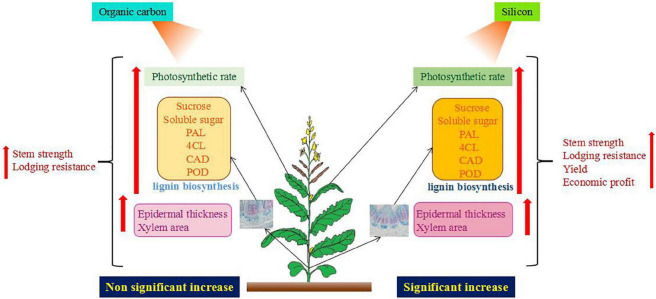
Effect of organic carbon and silicon fertilizer on plant characteristics of rapeseed.

At the same time, the key enzyme activities and genes of lignin synthesis were upregulated from the bolting stage to the green pod stage, and the treatment of silicon fertilizer was higher than that of organic carbon fertilizer. This was confirmed in lignin content (Figures 4, 5). The lignin content of Jayou is higher than that of Chuannongyou. Therefore, it has a higher stalk bending strength. Lignin content was positively correlated with PAL, 4CL, CAD, and POD enzyme activities. The results showed that higher PAL, 4CL, CAD, and POD enzyme activities were the enzymatic basis for promoting lignin accumulation in rapeseed stem. Regulating enzyme activity can increase lignin accumulation in rapeseed stalk and improve stalk strength. The increase of lignin content is achieved by increasing the activity of the lignin biosynthesis enzymes and gene expression during lignin synthesis (Hussain et al., 2021). The bolting stage is the key stage of stem development and lignin accumulation in rape. Organic carbon and silicon fertilizer up regulate the expression of lignin synthesis genes. The changing trend of PAL, 4CL, CAD, and POD genes was similar to that of the four enzymes. It was previously reported that silicon application significantly increased the transcription of PAL and 4CL, which enhanced the biosynthesis of monolignols (Fleck et al., 2011). The previous results of Sprayed silicon fertilizer solution on the leaves at the seedling stage of rapeseed promoted Lignin metabolism in the stalk. They improved the expression level of lignin synthesis genes (PAL, 4CL, and CAD) and lignin content (Kuai et al., 2017). This is consistent with the results of our study (Table 3). It was also stated that lignin metabolism plays an essential role in stem lodging resistance in maize (Kamran et al., 2018b), wheat (Kong et al., 2013), and other crops. Our data indicated that the carbohydrates, lignin, enzymatic activity, and gene expression levels were higher in the CK + OC + Si than CK + Si treatment, but there was no significant difference between the two treatments. Moreover, it can be concluded that organic carbon and silicon fertilizer have similar ways to affect lignin metabolism, but the effect of silicon is stronger than that of organic carbon (Figure 9).

### Anatomical Characteristics of Stem

The thickness of the stem mechanical tissue layer and vascular bundle sheath cells is the primary source of stem strength (Zhang et al., 2016a). In this study, four fertilizer treatments had significant effects on the internal tissue structure of rapeseed stem. Cortical thickness, vascular bundle area, mechanical layer thickness, and xylem area increased significantly and positively correlated with stem bending strength, indicating that these tissues play an important role in forming stem strength. The changing trend of the two varieties is the same, and these positive changes are conducive to enhancing the mechanical strength of the stem. Earlier, the vascular bundle area of wheat varieties with low bending strength was lower than that of high bending strength varieties. The mechanical tissue thickness could explain 99% of stalk lodging (Wang et al., 2006; Kong et al., 2013). The xylem area plays an important role in the stem strength of soybean (LIU et al., 2019). In comparison to CK + OC treatment, we revealed that applying CK + Si substantially increased the epidermal thickness and xylem area, which is also supported by the above studies. However, it was found that there was no significant effect on the stem cortex thickness of the two varieties under different fertilizer treatments.

### Yield and Economic Analysis

The increase in yield can be attributable to a combination of factors, including improved photosynthetic efficacy and increased nutrient absorption. Previous studies reported that the increase of plant biomass was linearly and positively correlated with nutrient absorption and photosynthetic rate (Guo et al., 2019). Organic carbon fertilizer is often used to improve soil properties and promote root development and microbial reproduction (Yao et al., 2021). The previous study of Cai et al. (2019) reported that the addition of organic carbon could increase the availability of nutrients by improving soil physical and chemical properties. Silicon plays a dynamic role in plant growth and development (Kim et al., 2017). The application of Si increased the number of pods per plant, the number of seeds in each pod, and the weight of 100 seeds, which was reported by a previous study (Artyszak, 2018). In 2005–2006, studies conducted in China on the application of Si to the soil revealed a 7.5–13.6% increase in soybean yield (Liang et al., 2015). It has also been reported that silicon may promote the expression of genes encoding nitrate transporters (*NRT* family) in roots and improve nitrogen absorption (Gottardi et al., 2012). Other studies have shown that silicon can also alter physical and chemical properties (e.g., soil exchange capacity) and biological properties of the soil (e.g., increasing the number of nitrogen-fixing microbes) and promote the uptake and accumulation of large amounts of nutrients and micronutrients by plants (Matichenkov and Bocharnikova, 2001). A recent study by Haddad et al. (2018). Showed that the beneficial effect of silicon on low nitrogen rape seedlings under short-term hydroponic conditions was surprising. The yield of CK + OC + Si treatment was the highest in this investigation, but there was no significant difference between CK + Si treatment and CK + OC + Si treatment. This may be because the stimulation of organic carbon fertilizer and silicon fertilizer on the growth and nutrition of rapeseed has reached saturation which can be proved by photosynthetic rate and stem carbohydrate. This result is consistent with that observed by Laîné et al. (2019). From the results, we believe that photosynthetic performance may be an important reason for the lower yield of CK + OC treatment than CK + Si and CK + OC + Si treatment. The significant effect of silicon fertilizer on yield may suggest that the use of nitrogen fertilizer can be reduced without reducing rapeseed yield, which needs further research. Economic analysis of this study revealed that the addition of organic carbon fertilizer had no significant effect on yield growth, resulting in a decrease in net income. Due to the substantial increase in yield achieved with silicon fertilization, the final net revenue increased by 475.13 CNY Ha-1. (average value of two varieties) compared with conventional fertilization and combined the application of carbon and silicon fertilization reduces the net income due to the higher price of fertilizers (Table 6). Therefore, considering the economic benefits, we recommend that applying silicon fertilizer alone is a better choice. This may promote the future sale and use of silicon fertilizers in China, especially with the national promotion of a mechanistic and intensive mode of cultivation.

## Conclusion

Considering the outcomes of the current study, we concluded that both organic carbon and silicon fertilizers had improved the lignin content and increased the mechanical strength of rapeseed stem via enhancing the lignin accumulation, lignin biosynthesis enzymes, and their related genes. It was also found that stem bending strength was significantly associated with lignin content. Organic carbon and silicon fertilizers had similar effects on improving lignin content. However, the impact of silicon fertilizer was better than organic carbon and its mixed fertilizer. In comparing both genotypes, Jayou exhibited a higher value of lodging resistance index and lignin content under all treatments. The results demonstrated that the silicon application significantly enhanced the yield and economic efficiency while organic carbon had no significant effect on the yield. Our findings also contribute to a better understanding of how alone and coordinated applications of organic carbon and silicon fertilizer affect rapeseed stem strength, lignin metabolism, and physiological mechanisms, as well as serve as a baseline for future research.

## Data Availability Statement

The raw data supporting the conclusions of this article will be made available by the authors, without undue reservation.

## Author Contributions

Y-CW and YH: conceptualization. YH and HJ: methodology, formal analysis, and investigation. YH, XP, and F-FZ: formal analysis and investigation. YH, HJ, AG, and HC: writing–original draft preparation. YH, MB, HJ, and MA: writing–review and editing. Y-CW: supervision. All authors contributed to the article and approved the submitted version.

## Conflict of Interest

The authors declare that the research was conducted in the absence of any commercial or financial relationships that could be construed as a potential conflict of interest.

## Publisher’s Note

All claims expressed in this article are solely those of the authors and do not necessarily represent those of their affiliated organizations, or those of the publisher, the editors and the reviewers. Any product that may be evaluated in this article, or claim that may be made by its manufacturer, is not guaranteed or endorsed by the publisher.

## Footnotes

1
http://ycz.100ppi.com/
